# Correcting the literature: Improvement trends seen in contents of retraction notices

**DOI:** 10.1186/s13104-018-3576-2

**Published:** 2018-07-17

**Authors:** Evelyne Deculllier, Hervé Maisonneuve

**Affiliations:** 1 Pôle IMER, Unité de Recherche Clinique, Hospices Civils de Lyon, Lyon, France; 2 Université Lyon 1, Lyon, France; 3 Paris, France

**Keywords:** Publication practices, Retraction of publication, Scientific misconduct, Guidelines

## Abstract

**Objective:**

To analyse retraction notices from 2016 and compare their quality to the 2008 notices.

**Results:**

From 146 retractions retrieved, only 123 were included, of which, a clear reason for retraction was available for 122 (99.2%) and no reason was given for one (0.8%). The main reasons for retraction were mistakes 26.0% (*n* = 32), fraud 26.0% (*n* = 32), plagiarism 20.3% (*n* = 25), and overlap 8.1% (*n* = 10). In 100 (81.3%) cases, a mention of retraction was available on the original paper, in 15 (12.2%) there was no mention of retraction, and 8 (6.5%) papers were deleted. Compared to the previous cohorts, management of retraction has improved because 99.2% provided a clear reason, and 81.3% of original articles were available with a mention of the retraction.

**Electronic supplementary material:**

The online version of this article (10.1186/s13104-018-3576-2) contains supplementary material, which is available to authorized users.

## Introduction

It has been proven than proportion of retraction in Pubmed has increased tenfold between 1980s and 2005–2009 [1]. When looking beyond Pubmed, retractions are also growing: According to the RetractionWatch database (under formation yet), as on 6 February 2018, there were 1203 retractions in 2015, 1355 in 2016, and 1137 in 2017 (http://retractiondatabase.org).

There are several reasons for authors and/or editors to retract papers. According to studies, different classifications of retraction have been proposed. Over time, in the literature, the 2013 prediction of Smith, the then British Medical Journal editor, was achieved, with ‘fraud’ and ‘error’ cited as the two main reasons for retracting [2].

There is a need to correct the literature, and we applaud the Journal of the American Medical Association policy [3] since it uses retractions for pervasive errors and scientific or research misconduct [3]. In 2012, a detailed policy for retraction existed for 65% (*n* = 147) of the top scientific journals ranked by impact factor [4]. Too many journals err in not correcting the literature. The quality of retraction notices has been challenged since studies observed that 3–18% of notices had no clear reason for retraction [5]. The RetractionWatch website publishes comments daily on retraction and on poorly written retractions. Most of the retractions are unclear and/or too short. Moreover, retracted papers do not always follow good practices (for example, make a specific mention of the availability of the original paper).

We previously analysed 244 retractions notices published in 2008 [6]. Formal retraction notices could not be retrieved for 9 of them. Of the 235 retractions available (96%), the reason was not explicit in 21 articles (9%). The most cited reasons were mistakes (28%), plagiarism (20%), fraud (14%), and overlap (11%). The original paper or its location was mentioned in 233 retractions (95%). Of these, 22% were available with no mention of the retraction.

No study on retraction was published after 2010, and hence, there is lack of information on whether the quality of retraction notices has improved over time. Our objective was to analyse retraction notices from 2016 and compare them to the 2008 notices.

## Main text

### Methods

To allow comparisons, we used the same methodology and classification, which is explained below, as in our previous article [5]. We included all the complete retraction notices published between 1 March 2016 and 30 June 2016. We searched Medline on 1 February 2017 and retrieved the notices of retraction and the original articles. For each retraction, we examined the reason for retraction and categorised it if available. Using the same search strategy, we retrieved a new kind of notice, which was applicable only to Elsevier’s policy and was labelled ‘Withdrawn’ (https://www.elsevier.com/about/our-business/policies/article-withdrawal). These notices did not concern published papers, but were rather in-press articles, and they were therefore excluded.

We also retrieved the country of affiliation of the first author and the mention of retraction on the original article. The two authors read all the notices and appraised them. When discrepancies were observed, consensus was obtained by a common reading. The main outcome measure was the availability of a clear reason for retraction.

### Results

Of the total 146 retractions retrieved (complete list available in Additional file 1), 6 were excluded (1 indexation error, 3 retracting multiple articles, 1 retraction of a newsletter, and 1 temporary removal before publication). We discarded 16 ‘withdrawn’ notices. One notice was not available. The final population consisted of 123 retractions.

A clear reason for retraction was available for 122 (99.2%), and no reason was given in one case (0.8%). The main reasons were mistakes 26.0% (*n* = 32), fraud 26.0% (*n* = 32), plagiarism 20.3% (*n* = 25), and overlap 8.1% (*n* = 10), Table 1. The frequency of fraud came down to 9% if image manipulations were not included. Countries of origin of the first authors were China (*n* = 29), United States of America (*n* = 23), India (*n* = 14), Spain (8), Brazil (7), Italy (6); 22 countries had 4 or less retractions. A mention of retraction was available on the original paper for 81.3% (*n* = 100); 12.2% (*n* = 15) were available without mention, and 6.5% (*n* = 8) of original papers were deleted.

**Table 1:** Reason for retraction among the 123 retractions

Fraud	Falsified data, fabricated data	32
Mistakes/inconsistent data	Mistakes concerning data found in the paper raised by the author(s)/confirmed doubt over data raised by others	32
Plagiarism	Publication of data or text already published by others or self-plagiarism^a^	25
Overlap	Multiple publication of same data	10
Property or legal concerns	Publication of elements without obtaining permission	3
Ethics	Concerns on the ethical validation of the research	2
Authorship	Disputed authorship	0
Editor	Production or administrative error	4
Others	*Peer review process, quality of research, reproducibility*	14

No reason provided for 1 retraction

^a^In the previous publication, self plagiarism was categorized as overlap

### Discussion

Compared to the previous cohorts, management of retraction has improved with 99.2% providing a clear reason and 81.3% of the original articles still available with a mention of the retraction.

We believe that management of retraction has improved with time. In 2008, we observed that 21/235 (9%) notices did not state the reason for retracting the paper. We used the same methods with the 2016 sample and observed that 0.8% of 123 notices mentioned no reasons for retraction. Before 2010, studies observed that 3–18% of notices showed no reason for retraction. Moreover, we found that mention of retraction was available on 81.3% of retracted articles, this is also an improvement compared to the 60% found in 2008.

Our PubMed literature search was exhaustive; it was conducted over a 4-month period and represents a good sample for 2016, which allows for comparison with our 2008 sample (244 notices retrieved).

The reasons for retraction in the samples of 2008 and 2016 were relatively similar. A slight difference was observed for the reason ‘fraud’, which accounted for 14% of the retraction reasons in 2008 and 26% in 2016. We hypothesise that this difference is due to the high presence of image manipulation. Indeed, during the review process, the authors felt that image manipulations were often cited, and therefore a specific variable was created. However, since this variable was not specifically searched in 2008, we cannot be certain that the difference is only due to image manipulation.

The need to report better retraction persists, as reasons are sometimes difficult to classify. We proposed using a standard form, but this requires testing [7]. This form is sometimes used by small journal editors. Retractions that are issued by large publishers are written and/or proofread by lawyers and do not comply with an open standard form. Practices of top-tier journals have improved, and most editors are willing to improve the quality of the literature.

## Limitations

The study period was short (4 months), however it was sufficient to describe the trend in retractions.

## Additional file

**Additional file 1.** Retractions list. References of all identified retractions classified according to status (exclusion; not found/withdrawn; included).
