# Supplementary material for: Correcting the literature: Improvement trends seen in contents of retraction notices
Source: BMC Res Notes. 2018 Jul 17;11:490. doi: 10.1186/s13104-018-3576-2 (PMC6050656; doi:10.1186/s13104-018-3576-2)
Supplement: Supplementary file 1 — Additional file 1. Retractions list. References of all identified retractions classified according to status (exclusion; not found/withdrawn; included). [file 13104_2018_3576_MOESM1_ESM.docx]

**EXCLUSION (n=6)**

- Bevilacqua L, Liani G, Castronovo G, Costantinides F. Clinical and spectrophotometric evaluation after chlorhexidine use in periodontal flap surgery: A prospective randomized clinical trial. Am J Dent. 2016;29(2):75-80. Epub 2016/06/15.
- Notice of Retraction. Anesth Analg. 2016;122(5):1730. Epub 2016/04/23.
- Lee VH. TEMPORARY REMOVAL: Professor A.T. Florence: A Towering Figure in Pharmaceutics. Int J Pharm. 2016. Epub 2016/05/07.
- Retracted Papers from the Journal "Medical Archives". Med Arch. 2016;70(3):243. Epub 2016/09/01.
- Doki Y. Retraction Note: Notice of formal retraction of articles by Dr. Akihiro Cho. Surg Today. 2016;46(5):631. Epub 2016/02/18.
- WITHDRAWN: World Apheresis Association Newsletter: April 1, 2016. Transfus Apher Sci. 2016. Epub 2016/06/05.

**WITHDRAWN/NOT FOUND (n=17)**

- Zhang N, He L, Wang J, Guo Y, Liu Y, Kong Y, et al. WITHDRAWN: Chronic intermittent hypobaric hypoxia attenuates radiation-induced heart damage in rats. Biochem Biophys Res Commun. 2016. Epub 2016/11/05.
- Zhang P, Zhou HX, Yang MX, Wang Y, Cao WM, Lu KF, et al. WITHDRAWN: miR-543 promotes proliferation and invasion of non-small cell lung cancer cells by inhibiting PTEN. Biochem Biophys Res Commun. 2016. Epub 2016/04/05.
- Li X, Zhang Y, Chen Y, He W, Wan H, Zhang L, et al. WITHDRAWN: Discovery of SHR1977: A highly potent and selective ROMK inhibitor. Bioorg Med Chem Lett. 2016. Epub 2016/07/06.
- WITHDRAWN: 44th Biennial American Cytogenetics Conference. Cancer Genet. 2016. Epub 2016/08/27.
- WITHDRAWN: 2016 American Cytogenetics Conference. Cancer Genet. 2016. Epub 2016/08/27.
- WITHDRAWN: Scientific Program. Cancer Genet. 2016. Epub 2016/08/27.
- WITHDRAWN: 2016 Recipient ACC Distinguished Cytogeneticist Award: Kathleen W. Rao, PhD, FACMG. Cancer Genet. 2016. Epub 2016/08/27.
- Yahya S, Benghiat H, Nightingale P, Tiffany M, Sanghera P, Hartley A. WITHDRAWN: Does Dose to an Oral Mucosa Organ at Risk Predict the Duration of Grade 3 Mucositis after Intensity-modulated Radiotherapy for Oropharyngeal Cancer? Clin Oncol (R Coll Radiol). 2016. Epub 2016/05/03.
- Li J, Liu LS, Fong S, Wong RK, Mohammed S, Fiaidhi J, et al. WITHDRAWN: Adaptive Swarm Balancing Algorithms for rare-event prediction in imbalanced healthcare data. Comput Med Imaging Graph. 2016. Epub 2016/05/30.
- Min X, Liu L, He Y, Gong X, Fong S, Xu Q, et al. WITHDRAWN: Benchmarking swarm intelligence clustering algorithms with case study of medical data. Comput Med Imaging Graph. 2016. Epub 2016/09/27.
- Tunholi-Alves VM, Tunholi VM, Teixeira Santos A, Dos Santos Bonfim TC, da Silva Garcia J, Maldonado A, Jr., et al. WITHDRAWN: Alterations in the mitochondrial physiology of Biomphalaria glabrata (Mollusca: Gastropoda) after experimental infection by Angiostrongylus cantonensis (Nematoda: Metastongylidae). Exp Parasitol. 2016. Epub 2016/06/01.
- Aras K, Burton B, Swenson D, MacLeod R. WITHDRAWN: Corrigendum to 'sensitivity of epicardial electrical markers to acute ischemia detection'. J Electrocardiol. 2016. Epub 2016/05/29.
- Ho AG, Gowda AL, Wiater JM. WITHDRAWN: Stemless humeral component in total shoulder arthroplasty: 2-year age-related clinical outcomes. J Shoulder Elbow Surg. 2016. Epub 2016/04/20.
- Sneve MH, Grydeland H, Amlien IK, Langnes E, Walhovd KB, Fjell AM. WITHDRAWN: Decoupling of large-scale brain networks supports the consolidation of durable episodic memories. Neuroimage. 2016. Epub 2016/05/25.
- Retraction Announcement. Pak J Med Sci. 2016;32(3):795. Epub 2016/07/05.
- Vaquerizo B, Larman M, Li CH, Lasa G, Fuertes M, Moreno R. WITHDRAWN: Use of Repositionable Transcatheter Aortic Valve Systems in Past Mechanical Prosthetic Mitral Valve Recipients. Rev Esp Cardiol (Engl Ed). 2016. Epub 2016/06/09.
- Paik HK, Ahn SS, Kim JY, Ryu DS, Kim KH, Chin DK, et al. TEMPORARY REMOVAL: Characteristics of patients with herniated discs at the cervicothoracic junction. Spine J. 2016. Epub 2016/05/10.

**INCLUDED (n=123)**

- Retraction. Am J Physiol Renal Physiol. 2016;310(11):F1423. Epub 2016/06/09.
- miR-128 modulates hepatocellular carcinoma by inhibition of ITGA2 and ITGA5 expression [Retraction]. Am J Transl Res. 2016;8(4):1919. Epub 2016/05/18.
- Retraction notice to "Localized surface plasmon resonance-based DNA detection in solution using gold-decorated superparamagnetic Fe3O4 nanocomposite" [Anal. Biochem. 465 (2014) 156-163]. Anal Biochem. 2016;501:82. Epub 2016/04/12.
- Origin, Reality, and Clinical Importance of the Arcade of Struthers, an Anatomic Study: Retraction. Ann Plast Surg. 2016;76(4):473. Epub 2016/03/10.
- Notice of Retraction. Arterioscler Thromb Vasc Biol. 2016;36(5):e52. Epub 2016/04/29.
- Elnoamany H. Retraction: Sensitivity of Pyramidal Signs in Patients with Cervical Spondylotic Myelopathy. Asian Spine J. 2016;10(2):406. Epub 2016/04/27.
- Retraction: Myostatin is a novel tumoral factor that induces cancer cachexia. Biochem J. 2016;473(8):1111. Epub 2016/04/10.
- BioMed Research I. Retracted: How to Perfuse: Concepts of Cerebral Protection during Arch Replacement. Biomed Res Int. 2016;2016:7147584. Epub 2016/05/05.
- Amato B, Compagna R, Corte GA, Martino G, Bianco T, Coretti G, et al. Retraction Note: Peripheral blood mono-nuclear cells implantation in patients with peripheral arterial disease: a pilot study for clinical and biochemical outcome of neoangiogenesis. BMC Surg. 2016;16(1):24. Epub 2016/04/29.
- Iacono F, Prezioso D, Illiano E, Ruffo A, Romeo G, Amato B. Retraction Note: Observational study: daily treatment with a new compound "tradamixina" plus serenoa repens for two months improved the lower urinary tract symptoms. BMC Surg. 2016;16(1):23. Epub 2016/04/29.
- Prezioso D, Iacono F, Di Lauro G, Illiano E, Romeo G, Ruffo A, et al. Retraction Note: Stress urinary incontinence: long-term results of laparoscopic Burch colposuspension. BMC Surg. 2016;16(1):26. Epub 2016/04/29.
- Rocco N, Rispoli C, Pagano G, Ascione S, Compagna R, Danzi M, et al. Retraction Note: Undertreatment of breast cancer in the elderly. BMC Surg. 2016;16(1):25. Epub 2016/04/29.
- Retraction: 'rhBNP therapy can improve clinical outcomes and reduce in-hospital mortality compared with dobutamine in heart failure patients: a meta-analysis' by Ming-Yi Lv, Shu-Ling Deng and Xiao-Feng Long. Br J Clin Pharmacol. 2016;81(5):1005. Epub 2016/04/19.
- Liu G, Wu Q, Song X, Zhang J. Retraction Note: Psoriasin (S100A7) is a novel biomarker for lung squamous cell carcinoma in humans. Cancer Cell Int. 2016;16:40. Epub 2016/06/03.
- Retraction: High-Avidity T Cells Are Preferentially Tolerized in the Tumor Microenvironment. Cancer Res. 2016;76(8):2491. Epub 2016/03/26.
- Retraction: Immunity to Murine Prostatic Tumors: Continuous Provision of T-Cell Help Prevents CD8 T-Cell Tolerance and Activates Tumor-Infiltrating Dendritic Cells. Cancer Res. 2016;76(8):2490. Epub 2016/03/26.
- Retraction statement: 'Formin-like2 regulates Rho/ROCK pathway to promote actin assembly and cell invasion of colorectal cancer' by Yuanfeng Zeng, Huijun Xie, Yudan Qiao, Jianmei Wang, Xiling Zhu, Guoyang He, Yuling Li, Xiaoli Ren, Feifei Wang, Li Liang and Yanqing Ding. Cancer Sci. 2016;107(7):1060. Epub 2016/07/16.
- Notice of Retraction. Carcinogenesis. 2016;37(5):530. Epub 2016/02/28.
- Case Reports In O, Gynecology. Retracted: Prenatal Diagnosis of Concurrent Achondroplasia and Klinefelter Syndrome. Case Rep Obstet Gynecol. 2016;2016:4363897. Epub 2016/05/31.
- Bill A, Schmitz A, Albertoni B, Song JN, Heukamp LC, Walrafen D, et al. Retraction Notice to: Cytohesins Are Cytoplasmic ErbB Receptor Activators. Cell. 2016;165(5):1293. Epub 2016/05/21.
- Shin YJ, Riew TR, Park JH, Pak HJ, Lee MY. Retraction Note To: Expression of SOCS2 mRNA and protein in the ischemic core and penumbra after transient focal cerebral ischemia in rats. Cell Tissue Res. 2016;364(3):681. Epub 2016/05/01.
- Retraction Note to: Subependymal giant cell astrocytoma: current concepts, management, and future directions. Childs Nerv Syst. 2016;32(4):761. Epub 2016/02/29.
- Notice of Withdrawal: "Comparison of fit accuracy between Procera custom abutments and three implant systems" by Tiago de Morais Alves da Cunha, Roberto Paulo Correia de Araujo, Paulo Vicente Barbosa da Rocha and Rosa Maria Pazos Amoedo. Clin Implant Dent Relat Res. 2016;18(2):421. Epub 2016/02/10.
- Retraction Note: Drying of a plasmid containing formulation: chitosan as a protecting agent. Daru. 2016;24:11. Epub 2016/04/28.
- Carvalho CR, Thirone AC, Gontijo JA, Velloso LA, Saad MJ. Statement of Retraction. Effect of Captopril, Losartan, and Bradykinin on Early Steps of Insulin Action. Diabetes 1997;46:1950-1957. DOI: 10.2337/diab.46.12.1950. Diabetes. 2016;65(4):1128. Epub 2016/05/22.
- Duca FA, Sakar Y, Lepage P, Devime F, Langelier B, Dore J, et al. Statement of Retraction. Replication of Obesity and Associated Signaling Pathways Through Transfer of Microbiota From Obese-Prone Rats. Diabetes 2014;63:1624-1636. DOI: 10.2337/db13-1526. Diabetes. 2016;65(5):1447. Epub 2016/05/22.
- Flores MB, Fernandes MF, Ropelle ER, Faria MC, Ueno M, Velloso LA, et al. Statement of Retraction. Exercise Improves Insulin and Leptin Sensitivity in Hypothalamus of Wistar Rats. Diabetes 2006;55:2554-2561. DOI: 10.2337/db05-1622. Diabetes. 2016;65(4):1127-8. Epub 2016/05/22.
- Oliveira AG, Carvalho BM, Tobar N, Ropelle ER, Pauli JR, Bagarolli RA, et al. Statement of Retraction. Physical Exercise Reduces Circulating Lipopolysaccharide and TLR4 Activation and Improves Insulin Signaling in Tissues of DIO Rats. Diabetes 2011;60:784-796. DOI: 10.2337/db09-1907. Diabetes. 2016;65(4):1124-5. Epub 2016/05/22.
- Tsukumo DM, Carvalho-Filho MA, Carvalheira JB, Prada PO, Hirabara SM, Schenka AA, et al. Statement of Retraction. Loss-of-Function Mutation in Toll-Like Receptor 4 Prevents Diet-Induced Obesity and Insulin Resistance. Diabetes 2007;56:1986-1998. DOI: 10.2337/db06-1595. Diabetes. 2016;65(4):1126-7. Epub 2016/05/22.
- Frush S, Li Z, Potts EN, Du W, Eu JP, Garantziotis S, et al. Retraction: "The Role of the Extracellular Matrix Protein Mindin in Airway Response to Environmental Airways Injury". Environ Health Perspect. 2016;124(4):A69. Epub 2016/04/02.
- Paulik LB, Donald CE, Smith BW, Tidwell LG, Hobbie KA, Kincl L, et al. Retraction of "Impact of Natural Gas Extraction on PAH Levels in Ambient Air". Environ Sci Technol. 2016;50(14):7936. Epub 2016/06/30.
- Tidwell LG, Allan SE, O'Connell SG, Hobbie KA, Smith BW, Anderson KA. Retraction of "Polycyclic Aromatic Hydrocarbon (PAH) and Oxygenated PAH (OPAH) Air-Water Exchange during the Deepwater Horizon Oil Spill". Environ Sci Technol. 2016;50(14):7935. Epub 2016/06/30.
- No more pain upon Gq-protein-coupled receptor activation: role of endocannabinoids. Eur J Neurosci. 2016;43(7):991. Epub 2016/04/05.
- A dietary polyphenol resveratrol acts to provide neuroprotection in recurrent stroke models by regulating AMPK and SIRT1 signaling, thereby reducing energy requirements during ischemia. Eur J Neurosci. 2016;43(7):990. Epub 2016/04/05.
- Retraction. Eur J Orthod. 2016;38(2):225. Epub 2016/03/10.
- microRNA-106a induces the proliferation and apoptosis of glioma cells through regulating JNK/MAPK pathway. Eur Rev Med Pharmacol Sci. 2016;20(7):1221. Epub 2016/04/22.
- Retraction notice to "Ski diminishes TGF-beta1-induced myofibroblast phenotype via up-regulating Meox2 expression". Exp Mol Pathol. 2016;100(2):362. Epub 2016/04/07.
- Retraction: Molecular structure and target recognition of neuronal calcium sensor proteins. Front Mol Neurosci. 2016;9:38. Epub 2016/06/01.
- Retraction: Mechanism of Action of the Novel Nickel(II) Complex in Simultaneous Reactivation of the Apoptotic Signaling Networks Against Human Colon Cancer Cells. Front Pharmacol. 2016;7:199. Epub 2016/07/23.
- Retraction: Water hyacinth: a possible alternative rate retarding natural polymer used in sustained release tablet design. Front Pharmacol. 2016;7:141. Epub 2016/06/09.
- Retraction: The Carboxy-terminus of BAK1 regulates kinase activity and is required for normal growth of Arabidopsis. Front Plant Sci. 2016;7:960. Epub 2016/07/23.
- Retraction notice to "Increased Risk of Stomach and Esophageal Malignancies in People With AIDS": Gastroenterology 2012;143:943-950.e2. Gastroenterology. 2016;150(4):1048. Epub 2016/03/27.
- Retraction notice to "The identification of loci for polydactyly in chickens using a genome-wide association study" [GENE 568/2 (2015) 176-180]. Gene. 2016;584(2):195. Epub 2016/04/07.
- Retraction. Hypertension. 2016;67(6):e23. Epub 2016/05/14.
- Retraction: Surgical reconstruction or prosthetic rehabilitation following orbital exenteration: The clinician's dilemma. Indian J Plast Surg. 2016;49(2):298. Epub 2016/11/12.
- Retraction. Int J Cancer. 2016;138(8):2050. Epub 2016/02/09.
- Post-dural puncture headache [Retraction]. Int J Gen Med. 2016;9:173. Epub 2016/06/23.
- International Journal Of Molecular Sciences Editorial O. Retraction: Zihan Xu, et al. Tanshinone IIA Pretreatment Renders Free Flaps against Hypoxic Injury through Activating Wnt Signaling and Upregulating Stem Cell-Related Biomarkers. Int. J. Mol. Sci. 2014, 15, 18117-18130. Int J Mol Sci. 2016;17(5). Epub 2016/05/24.
- Retraction notice to "Urinary sodium or potassium excretion and blood pressure in adults of Shandong province, China: preliminary results of the SMASH project": [J Am Soc Hypertens 9 (2015) 754-762]. J Am Soc Hypertens. 2016;10(5):472. Epub 2016/05/08.
- Retraction. J Appl Oral Sci. 2016;24(3):308. Epub 2016/07/08.
- Astrocyte resilience to oxidative stress induced by insulin-like growth factor I (IGF-I) involves preserved AKT (protein kinase B) activity. J Biol Chem. 2016;291(23):12039. Epub 2016/06/05.
- Presenilin-1 interacts with plakoglobin and enhances plakoglobin-Tcf-4 association. IMPLICATIONS FOR THE REGULATION OF beta-CATENIN/Tcf-4-DEPENDENT TRANSCRIPTION. J Biol Chem. 2016;291(21):11464. Epub 2016/05/27.
- Regulation of beta-catenin structure and activity by tyrosine phosphorylation. J Biol Chem. 2016;291(21):11463. Epub 2016/05/27.
- beta-Catenin N- and C-terminal tails modulate the coordinated binding of adherens junction proteins to beta-catenin. J Biol Chem. 2016;291(21):11462. Epub 2016/05/27.
- Cytomegalovirus promoter up-regulation is the major cause of increased protein levels of unstable reporter proteins after treatment of living cells with proteasome inhibitors. J Biol Chem. 2016;291(17):8985. Epub 2016/04/30.
- Shulga N, Pastorino JG. Hexokinase II binding to mitochondria is necessary for Kupffer cell activation and is potentiated by ethanol exposure. J Biol Chem. 2016;291(24):12574. Epub 2016/06/12.
- Yi X, Wang P, Wang Z, Cai J, Hu M, Zhong G. Retraction Note to: Involvement of a Specific Chemosensory Protein from Bactrocera dorsalis in Perceiving Host Plant Volatiles. J Chem Ecol. 2016;42(5):461. Epub 2016/06/09.
- The RET/PTC-RAS-BRAF linear signaling cascade mediates the motile and mitogenic phenotype of thyroid cancer cells. J Clin Invest. 2016;126(4):1603. Epub 2016/04/02.
- Retraction: 'Effects of an olmesartan/amlodipine fixed dose on blood pressure control, some adipocytokines and interleukins levels compared with olmesartan or amlodipine monotherapies' by G. Derosa, A. F. G. Cicero, A. Carbone, F. Querci, E. Fogari, A. D'Angelo and P. Maffioli. J Clin Pharm Ther. 2016;41(2):237. Epub 2016/04/10.
- Li X, Guo C, Gu J, Duan W, Zhao M, Ma C, et al. RETRACTION of: Overexpression of VP, a vacuolar H+-pyrophosphatase gene in wheat (Triticum aestivum L.), improves tobacco plant growth under Pi and N deprivation, high salinity, and drought. J Exp Bot. 2016;67(9):2913. Epub 2016/05/11.
- Retraction: Suicide trends in Upper Egypt. J Forensic Sci. 2016;61(3):879. Epub 2016/04/29.
- Retraction Notice. J Int Oral Health. 2016;8(5):650. Epub 2016/08/11.
- Retraction Note to: Aneuploidy analysis of non-pronuclear embryos from IVF with use of array CGH: a case report. J Mol Histol. 2016;47(2):101. Epub 2015/12/30.
- KDM3A confers metastasis and chemoresistance in epithelial ovarian cancer. J Mol Histol. 2016;47(2):103. Epub 2015/10/27.
- Cao F, Yin A, Wen G, Sheikh AM, Tauqeer Z, Malik M, et al. Retraction Note: Alteration of astrocytes and Wnt/beta-catenin signaling in the frontal cortex of autistic subjects. J Neuroinflammation. 2016;13(1):106. Epub 2016/05/15.
- Author-Initiated Retraction: Anderson et al, Attending Multiple Items Decreases the Selectivity of Population Responses in Human Primary Visual Cortex. J Neurosci. 2016;36(15):4404. Epub 2016/04/15.
- Author-Initiated Retraction: Donmez et al., SIRT1 Protects against alpha-Synuclein Aggregation by Activating Molecular Chaperones. J Neurosci. 2016;36(14):4138. Epub 2016/04/08.
- RETRACTION OF ARTICLE ON GROUNDS OF REDUNDANT/DUPLICATE PUBLICATION. J Pak Med Assoc. 2016;66(4):496. Epub 2016/04/29.
- Notice of Retraction: Sato Y, et al. Effect of Folate and Mecobalamin on Hip Fractures in Patients With Stroke: A Randomized Controlled Trial. JAMA. 2005;293(9):1082-1088. JAMA. 2016;315(22):2405. Epub 2016/06/04.
- Jang JY, Cai J, Kim J, Kyung J, Kim D, Choi EK, et al. Withdrawal: Specific nephrotoxicity and cardiotoxicity of BT-CAL(R), Sigma Anti-bonding Molecule Calcium Carbonate, in mice. Lab Anim Res. 2016;32(2):134. Epub 2016/07/07.
- Jun Ji, Dr Li Zhang, Yuan-Yuan Wu, Xiao-Yu Zhu, Su-Qing Lv, Xi-Zuo Sun, Induction of apoptosis by d-limonene is mediated by a caspase-dependent mitochondrial death pathway in human leukemia cells. December 2006;47(12):2617-2624. http://dx.doi.org/10.1080/00268970600909205. Leuk Lymphoma. 2016;57(5):1242. Epub 2016/04/21.
- Hu Jie, He Donghua, Xue Xingkui, Gao Liang, Wu Wenjun, Han Xiaoyan, Cai Zhen, Homoharringtonine-induced apoptosis of MDS cell line MUTZ-1 cells is mediated by the endoplasmic reticulum stress pathway, May 2007;48(5):964-977. http://dx.doi.org/10.1080/10428190701216360. Leuk Lymphoma. 2016;57(5):1241. Epub 2016/04/21.
- Peihua Luo, Meili Lin, Meihua Lin, Yiyu Chen, Bo Yang, Qiaojun He, Function of retinoid acid receptor a and p21 in all-trans-retinoic acid-induced acute T-lymphoblastic leukemia apoptosis. July 2009;50(7):1183-1189. http://dx/doi.org/10.1080/10428190902934936. Leuk Lymphoma. 2016;57(5):1243. Epub 2016/03/08.
- Wei Zhang, Bao-an Chen, Jun-fei Jin, You-ji He, and Yi-qi Niu, Involvement of c-Jun N-terminal kinase in reversal of multidrug resistance of human leukemia cells in hypoxia by 5-bromotetrandrine. November 2013;54(11):2506-2516. http://dx.doi.org/10.3109/10428194.2013.776681. Leuk Lymphoma. 2016;57(5):1244. Epub 2016/03/05.
- Adeniji AA, Rumak S, Oluwafemi RA. Retraction Note: Effects of replacing groundnut cake with rumen content supplemented with or without enzyme in the diet of weaner rabbits. Lipids Health Dis. 2016;15(1):101. Epub 2016/05/29.
- RETRACTED PAPERS FROM THE JOURNAL "MATERIA SOCIO-MEDICA". Mater Sociomed. 2016;28(3):239. Epub 2016/08/03.
- Retraction notice. Med Leg J. 2016;84(3):168. Epub 2016/05/08.
- Long-term Follow-up Results from PET/CT Surveillance after Surgical Resection of Lung Adenocarcinoma Manifesting as Ground-glass Opacity: Notice of Retraction. Medicine (Baltimore). 2016;95(15):e6038. Epub 2016/06/01.
- Confirmatory factor analysis for assessment of the menopausal representation questionnaire: Retraction. Menopause. 2016;23(6):e1. Epub 2016/05/21.
- Retraction Note to: TNF receptor-associated factor 6 regulates proliferation, apoptosis, and invasion of glioma cells. Mol Cell Biochem. 2016;415(1-2):207. Epub 2016/03/20.
- Statement of Retraction. Nat Prod Res. 2016;30(21):2505. Epub 2016/04/16.
- Abdul Ajees A, Volanakis JE, Narayana SV. Retraction: The structure of complement C3b provides insights into complement activation and regulation. Nature. 2016;532(7598):268. Epub 2016/01/05.
- Dai A, Liu X, Zhang Y, Han L, Zhu L, Ni H, et al. Retraction Note to: Up-Regulation of KPNB1 Involves in Neuronal Apoptosis Following Intracerebral Hemorrhage in Adult Rats. Neurochem Res. 2016;41(6):1505. Epub 2016/04/15.
- Retraction notice to "S14G-humanin Ameliorates Abeta 25-35-induced Behavioral Deficits by Reducing Neuroinflammatory Responses and Apoptosis in Mice" [Neuropeptides 42/5-6 (2008) 557-567]. Neuropeptides. 2016;56:125. Epub 2016/04/02.
- Cassani RS, Fassini PG, Silvah JH, Lima CM, Marchini JS. Retraction Note: Impact of weight loss diet associated with flaxseed on inflammatory markers in men with cardiovascular risk factors: a clinical study. Nutr J. 2016;15(1):59. Epub 2016/06/09.
- Upregulation of nucleostemin in colorectal cancer and its effects on cell malignancy [Retraction]. Onco Targets Ther. 2016;9:2625. Epub 2016/05/27.
- Editorial retraction. Oncoimmunology. 2016;5(5):e1173506. Epub 2016/07/29.
- Du WK, Hung HY, Kuo PC, Hwang TL, Shiu LC, Shiu KB, et al. Retraction of "Dragonbloodin A1 and A2: Flavan Trimers and Anti-inflammatory Principles from Sanguis Draconis". Org Lett. 2016;18(12):3042. Epub 2016/04/12.
- Pathology Research I. Retracted: Oral Carcinogenesis and Oral Cancer Chemoprevention: A Review. Patholog Res Int. 2016;2016:9267585. Epub 2016/07/20.
- Retraction: Mechanism of action of novel piperazine containing a toxicant against human liver cancer cells. PeerJ. 2016;4. Epub 2017/01/31.
- Retraction Note: RGD peptide-pegylated PLLA nanoparticles containing epirubicin hydrochloride exhibit receptor-dependent tumor trafficking in vitro and in vivo. Pharm Res. 2016;33(4):1050. Epub 2016/02/04.
- Retraction for Christensen CC, Antoszewski S, Maus E, et al. Establishing Reference Values for Infant Head Shape Using a Modified Version of Plagiocephalometry. Phys Ther. Epub ahead of print May 12, 2016. doi: 10.2522/ptj.20150366. Phys Ther. 2016;96(8):1299. Epub 2016/05/14.
- Dasgupta N, Biswas P, Kumar R, Kumar N, Bera B, Das S. Retraction Note to: Antioxidants and ROS scavenging ability in ten Darjeeling tea clones may serve as markers for selection of potentially adapted clones against abiotic stress. Physiol Mol Biol Plants. 2016;22(2):287. Epub 2016/07/21.
- Mukhtar S, Ahmad N, Khan MI, Anis M, Aref IM. Retraction Note to: influencing micropropagation in Clitoria ternatea L. through the manipulation of TDZ levels and use of different explant types. Physiol Mol Biol Plants. 2016;22(2):289. Epub 2016/07/21.
- Caricilli AM, Picardi PK, de Abreu LL, Ueno M, Prada PO, Ropelle ER, et al. Retraction: Gut Microbiota Is a Key Modulator of Insulin Resistance in TLR 2 Knockout Mice. PLoS Biol. 2016;14(5):e1002479. Epub 2016/05/24.
- Retraction: Genetically Engineered Synthetic Miniaturized Versions of Plasmodium falciparum UvrD Helicase Are Catalytically Active. PLoS One. 2016;11(6):e0158859. Epub 2016/07/01.
- Retraction: The Effect of Social Stress on Chronic Pain Perception in Female and Male Mice. PLoS One. 2016;11(5):e0156567. Epub 2016/05/26.
- Retraction: Ectopic Expression of a Maize Hybrid Down-Regulated Gene ZmARF25 Decreases Organ Size by Affecting Cellular Proliferation in Arabidopsis. PLoS One. 2016;11(5):e0155904. Epub 2016/05/14.
- Brass DM, Spencer JC, Li Z, Potts-Kant E, Reilly SM, Dunkel MK, et al. Retraction: Innate Immune Activation by Inhaled Lipopolysaccharide, Independent of Oxidative Stress, Exacerbates Silica-Induced Pulmonary Fibrosis in Mice. PLoS One. 2016;11(5):e0155388. Epub 2016/05/10.
- Hale LP, Kant EP, Greer PK, Foster WM. Retraction: Iron Supplementation Decreases Severity of Allergic Inflammation in Murine Lung. PLoS One. 2016;11(5):e0155387. Epub 2016/05/10.
- Ndiath MO, Mazenot C, Sokhna C, Trape JF. Retraction: How the Malaria Vector Anopheles gambiae Adapts to the Use of Insecticide-Treated Nets by African Populations. PLoS One. 2016;11(5):e0156196. Epub 2016/05/20.
- Ndiath MO, Sougoufara S, Gaye A, Mazenot C, Konate L, Faye O, et al. Retraction: Resistance to DDT and Pyrethroids and Increased kdr Mutation Frequency in An. gambiae after the Implementation of Permethrin-Treated Nets in Senegal. PLoS One. 2016;11(5):e0156195. Epub 2016/05/20.
- Watthanasurorot A, Jiravanichpaisal P, Liu H, Soderhall I, Soderhall K. Retraction: Bacteria-Induced Dscam Isoforms of the Crustacean, Pacifastacus leniusculus. PLoS Pathog. 2016;12(5):e1005630. Epub 2016/05/06.
- Retraction for Oh et al., Autophosphorylation of Tyr-610 in the receptor kinase BAK1 plays a role in brassinosteroid signaling and basal defense gene expression. Proc Natl Acad Sci U S A. 2016;113(27):E3987. Epub 2016/06/22.
- Retraction for Zhang et al., Stabilization of vaccines and antibiotics in silk and eliminating the cold chain. Proc Natl Acad Sci U S A. 2016;113(26):E3810. Epub 2016/06/22.
- Retraction for Dong et al., Deficient Smad7 expression: A putative molecular defect in scleroderma. Proc Natl Acad Sci U S A. 2016;113(15):E2208. Epub 2016/04/05.
- Shah M. RETRACTED: Auricular prosthesis fabrication using computer-aided design and rapid prototyping technologies. Prosthet Orthot Int. 2016;40(3):NP1. Epub 2013/10/10.
- Retraction Note to: Correlation of ESR, C3, C4, anti-DNA and lupus activity based on British Isles Lupus Assessment Group Index in patients of rheumatology clinic. Rheumatol Int (2010)30:1605-9. Rheumatol Int. 2016;36(5):749. Epub 2016/04/02.
- Palmer G, Hill JK, Brereton TM, Brooks DR, Chapman JW, Fox R, et al. Retraction of the Research Article: "Individualistic sensitivities and exposure to climate change explain variation in species' distribution and abundance changes". Sci Adv. 2016;2(4):e1600819. Epub 2016/07/08.
- Retraction: Novel piperazine core compound induces death in human liver cancer cells: possible pharmacological properties. Sci Rep. 2016;6:29056. Epub 2016/06/23.
- Xiang JF, Wang WQ, Liu L, Xu HX, Wu CT, Yang JX, et al. Retraction: Mutant p53 determines pancreatic cancer poor prognosis to pancreatectomy through upregulation of cavin-1 in patients with preoperative serum CA19-9 >/= 1,000 U/mL. Sci Rep. 2016;6:25115. Epub 2016/05/14.
- The Scientific World J. Retracted: Medical Dataset Classification: A Machine Learning Paradigm Integrating Particle Swarm Optimization with Extreme Learning Machine Classifier. ScientificWorldJournal. 2016;2016:7137054. Epub 2016/05/21.
- The Scientific World J. Retracted: An Automatic Web Service Composition Framework Using QoS-Based Web Service Ranking Algorithm. ScientificWorldJournal. 2016;2016:6902846. Epub 2016/05/21.
- The Scientific World J. Retracted: Dynamic Harmony Search with Polynomial Mutation Algorithm for Valve-Point Economic Load Dispatch. ScientificWorldJournal. 2016;2016:4953678. Epub 2016/05/21.
- The Scientific World J. Retracted: An Improved Differential Evolution Solution for Software Project Scheduling Problem. ScientificWorldJournal. 2016;2016:4835617. Epub 2016/05/21.
- The Scientific World J. Retracted: Differential Evolution Algorithm with Diversified Vicinity Operator for Optimal Routing and Clustering of Energy Efficient Wireless Sensor Networks. ScientificWorldJournal. 2016;2016:1520847. Epub 2016/05/21.
- Zeng W, Li S, Wang Z. Retraction: Linear model of a T-junction microdroplet generator for precise control of droplet size. Soft Matter. 2016;12(18):4274. Epub 2016/04/23.
- Retraction: Solid hemangioblastoma in the cerebellopontine angle: Importance of external carotid blood supply with regard to the probable site of origin and preoperative embolization. Surg Neurol Int. 2016;7:42. Epub 2016/04/30.
- Retraction of: Reversal of Spinal Cord Ischemia Following Endovascular Thoracic Aortic Aneurysm Repair with Hyperbaric Oxygen and Therapeutic Hypothermia, by Urquieta E, et al. Ther Hypothermia Temp Manag. DOI: 10.1089/ther.2015.0025. Ther Hypothermia Temp Manag. 2016;6(2):110. Epub 2016/06/11.
- Retraction of: Berg, et al., DOI: 10.1089/ten.tea.2012.0514. Tissue Eng Part A. 2016;22(9-10):829. Epub 2016/05/06.
- Retraction to platelets and their role in cancer evolution and immune system. Transl Lung Cancer Res. 2016;5(2):213. Epub 2016/05/18.
- Retraction. Vet Pathol. 2016;53(3):697. Epub 2015/06/06.
- Liu Z, Zhang W, Fan S, Wang L, Jiao L. Retraction Note: Changes in the electron paramagnetic resonance spectra of albumin-associated spin-labeled stearic acid as a diagnostic parameter of colorectal cancer. World J Surg Oncol. 2016;14(1):156. Epub 2016/06/04.
